# Transcranial Alternating Current Stimulation Alters Auditory Steady-State Oscillatory Rhythms and Their Cross-Frequency Couplings

**DOI:** 10.1177/15500594231179679

**Published:** 2023-06-12

**Authors:** Sara de la Salle, Joëlle Choueiry, Mark Payumo, Matt Devlin, Chelsea Noel, Ali Abozmal, Molly Hyde, Renée Baysarowich, Brittany Duncan, Verner Knott

**Affiliations:** 1 Clinical Neuroelectrophysiology and Cognitive Research, 580059 The Royal's Institute of Mental Health Research, Ottawa, ON, Canada; 2 Faculty of Medicine, School of Psychology, 6363 University of Ottawa, Ottawa, ON, Canada; 3 Department of Cellular and Molecular Medicine, 6363 University of Ottawa, Ottawa, ON, Canada; 4 School of Psychology, 6339 Carleton University, Ottawa, ON, Canada

**Keywords:** transcranial alternating current stimulation, electroencephalographic (EEG) oscillations, auditory steady-state response (ASSR), power, event-related spectral perturbation (ERSP), phase locking factor, theta-gamma phase–amplitude cross-frequency coupling (PAC), schizophrenia

## Abstract

Auditory cortical plasticity deficits in schizophrenia are evidenced with electroencephalographic (EEG)-derived biomarkers, including the 40-Hz auditory steady-state response (ASSR). Aiming to understand the underlying oscillatory mechanisms contributing to the 40-Hz ASSR, we examined its response to transcranial alternating current stimulation (tACS) applied bilaterally to the temporal lobe of 23 healthy participants. Although not responding to gamma tACS, the 40-Hz ASSR was modulated by theta tACS (vs sham tACS), with reductions in gamma power and phase locking being accompanied by increases in theta-gamma phase–amplitude cross-frequency coupling. Results reveal that oscillatory changes induced by frequency-tuned tACS may be one approach for targeting and modulating auditory plasticity in normal and diseased brains.

## Highlights

- 40-Hz ASSR attenuation implicates auditory cortical plasticity deficits.
- 40-Hz ASSR in response to tACS was assessed in healthy volunteers.
- Reductions in gamma power were seen only with low-frequency tACS.
- Reductions in phase locking were seen only with low-frequency tACS.
- Increases in theta-gamma coupling were seen only with low-frequency tACS.
- Findings suggest further study of tACS for modulating auditory neuroplasticity.

## Introduction

Deficits in low-level, sensory/perceptual processes in schizophrenia underly and contribute to cognitive impairment, a primary core feature of this disorder that is associated with poor psychosocial outcomes.^1–6^ Notably evident in the auditory domain and linked to structural abnormalities in the auditory cortex,^7^ disruptions in auditory sensory processes in schizophrenia are most effectively demonstrated using noninvasive electrophysiological measures, including electroencephalographically (EEG)-derived event-related potentials (ERPs)^8–12^ and steady-state oscillatory responses (SSRs).^13^

Elicited by temporally modulated auditory stimulation (amplitude modulated tones or click trains), the auditory steady-state response (ASSR) indexes the entrainment of EEG oscillations to the frequency (and phase) of the periodic auditory stimulus and, in the gamma frequency range (40-50 Hz) primarily reflects activity generated in the auditory cortex and associated neural pathways.^13^ In schizophrenia, disturbances in auditory synchronous activity are evident by reductions in ASSR total gamma power (also called event-related spectral perturbation [ERSP] which includes evoked power [phase locked to the stimulus] and induced power [not phase locked]) and phase (also called phase locking factor [PLF]) and are most consistent at 40 Hz.^14^ Unrelated to medication effects, appearing across all stages of the disorder,^15,16^ and associated with increased symptom severity and poor functioning,^17–19^ the 40-Hz ASSR abnormality in schizophrenia is correlated with reduced auditory cortex volume^20^ and is considered to reflect an auditory plasticity deficit that can be explained in part by aberrant N-methyl-D-aspartate glutamate receptor (NMDAR) activity.^14,21–24^ Considering its association with cognitive^25^ and real-world functioning^26^ in schizophrenia, the ASSR is positioned as a suitable translational biomarker^14,27^ for investigating disturbances in auditory sensory processing,^28^ synchrony and oscillatory activity and has translational implications for furthering our understanding of cognitive, clinical and functional improvements in schizophrenia and their response to novel treatments.^29–32^

As abnormalities in synchronized oscillations play a key role in the pathophysiology of schizophrenia,^33^ noninvasive transcranial electrical stimulation (tES) therapies such as transcranial alternating current stimulation (tACS) have a potential therapeutic role as they are used in an attempt to modulate cortical activity and plasticity in healthy and diseased brains.^34–37^ Administered noninvasively through scalp electrodes, low-intensity tACS entrains (synchronizes) endogenous neural oscillations to the specific frequency of the alternating current applied to stimulated cortical regions. Although not always showing consistent findings, preliminary studies in schizophrenia show that tACS improves negative and positive symptoms,^38–40^ and cognition (including attention, working memory, and emotional processing).^38,41–43^

In both animals and humans, tACS is used as a tool to understand the neural underpinnings of auditory processing, including the ASSR.^44,45^ Although a number of possible mechanisms of action of tACS have been proposed,^46,47^ matching of the external stimulating frequency with the endogenous activity which brings about the entrainment effects of tACS,^48^ ASSR studies typically employ tACS in the alpha (8-12 Hz) frequency range, the dominant frequency in the resting brain. Of the 2 studies carried out in healthy volunteers, both report reductions in the 40-Hz ASSR in response to alpha tACS applied bilaterally to the temporal lobe,^49,50^ while in the one study conducted in patients with schizophrenia, alpha tACS is reported to increase PLF of the 40-Hz ASSR.^51^ Although in healthy volunteers nonalpha tACS can influence auditory processes^45^ and endogenous oscillations in the auditory system,^44^ their effects on ASSR are mixed, with gamma tACS increasing ASSR power and phase locking,^52^ while theta tACS exerts no effect.^49^ Given the importance of abnormal gamma oscillations in NMDAR hypofunction models of schizophrenia,^53^ together with findings that the 40-Hz ASSR is a reliable pharmacodynamic biomarker for cortical NMDARs and auditory plasticity in schizophrenia,^14,21–24^ tACS may be a useful tool for investigating cause-effect oscillatory activity mediating normal and abnormal 40-Hz ASSR. The primary objective of this study is to examine the effects of gamma tACS on ERSP and PLF indices of the 40-Hz ASSR in healthy humans. It was hypothesized that gamma (vs sham) tACS would increase ERSP and PLF.

Different brain rhythms occurring simultaneously are known to interact and modulate each other,^54^ and hence, targeted (gamma) oscillations may reflect modulation by oscillations of other frequencies.^55^ Gamma oscillations are known to be modulated by neural oscillations in lower (eg, theta) frequency bands,^56–58^ evidencing phase–amplitude cross-frequency coupling (PAC),^59^ such that phase of the lower frequency oscillations modulate the amplitude of higher frequency oscillations.^57,58,60^ A secondary objective of this study is to assess the effects of theta tACS on the 40-Hz ASSR, and for both theta and gamma tACS, measure theta-gamma PAC in addition to ERSP and PLF. It was expected that theta (vs sham) tACS would enhance ERSP and PLF, and that increases in PAC would be observed with both theta and gamma (vs sham) tACS.

## Methods

### Participants

Twenty-seven right-handed males (18-30 years of age), all naïve to tACS, were recruited from the city population through online advertisements and from local universities. Only male participants were recruited to avoid potential confounding effects associated with gender differences and the menstrual cycle.^61^ Volunteers were screened with the Structured Clinical Interview for the Diagnostic and Statistical Manual of Mental Disorders (DSM-IV) Non-Patient Edition (SCID-I/NP)^62^ and in the Family Interview for Genetic Studies (FIGS)^63^ and in addition to reporting no personal or immediate family psychiatric diagnosis (Axis I disorders), study volunteers had to report no current medical illnesses, or neurological (including head injury) or substance abuse history. Volunteers were excluded if they reported excessive alcohol (more than 2 drinks/day) and caffeine (more than 5 cups/day) use or if they reported a smoking history (> 100 cigarettes in the past year). All participants provided written consent to the study.

### Design

Participants attended 3 testing sessions that were separated by a minimum of 3 days. Treatment sessions, including sham tACS, gamma tACS and theta tACS were embedded in a randomized, and repeated-measures, double-blind design.

### Procedures

Each of the 3 test sessions was conducted in the same manner between 9 am – 3 pm, prior to which participants were asked to abstain from food, alcohol, caffeine, nicotine, drugs, and beverages (except water) at least 12 h before arrival. Once they were seated in a reclining chair in a dimly-lit chamber, each session consisted of an identical sequence of procedures which included: (1) positioning of EEG electrodes on the scalp; (2) tACS administration; and (3) presentation of auditory stimulation for ASSR recording. Adverse events were assessed at the end of each session.

### Transcranial alternating current stimulation

tACS was applied with a battery-driven stimulator (NeuroConn, Ilmenay, Germany), which delivers a low amperage (1-2 mA), sinusoidal current alternating between the anode and cathode. The 5  × 7 cm rubber electrodes were superimposed in conductive 0.9% saline-soaked synthetic sponges. Relying on the 10-10 system for EEG electrode placement,^64^ the anode was placed over the left temporal hemisphere (electrode site T7) and the cathode was placed over the right temporal hemisphere (electrode site T8). Impedances were maintained below 10 kΩ. Theta tACS was set at 6 Hz and gamma tACS was set at 40 Hz.

Individual tACS intensity thresholds were assessed during the screening session by determining participants’ threshold for skin sensation. Theta and gamma tACS were applied starting at 1000 µA (1.0 mA). The amplitude was either increased or decreased in a stepwise manner in steps of 100 µA (0.1 mA) depending on the participant's self-report of the presence of skin sensations (eg, tingling and itching). The final tACS intensities administered during the testing sessions were 0.1 mA below each individual's threshold for skin sensation, a procedure used in previous work.^65^

In both active sessions, tACS was delivered at an individual threshold for 20 min (plus 10 s fade in/out). In the sham session, the threshold stimulation was turned on for 60 s (plus 10 s fade in) and then turned off for the remaining 19 min (with 10 s fade out). For half of the participants (selected at random), the sham frequency was 6 Hz and for the remaining half, it was set at 40 Hz.

### Auditory steady-state response

*Stimuli:* The ASSR paradigm was presented as participants watched a silent movie (Blue Planet by BBC Earth). Auditory stimulation consisted of 150 trains of 40 Hz, 1 ms clicks (trains being 500 ms duration with 1100 ms stimulus onset synchrony) presented binaurally at 80 dB sound pressure level (SPL) through headphones. The paradigm lasted approximately 3 min, during which they were asked to ignore the auditory stimulation and focus on the movie.

*Recordings:* EEG was recorded with Ag^+^/Ag^+^ Cl^−^ electrodes placed on the midline and left and right hemispheres of frontal (Fz, F3, and F4), central (Cz, C3, and C4), and parietal (Pz, P3, and P4) cortical areas, and on left (TP9) and right (TP10) mastoids. An electrode on the mid-forehead and nose tip served as ground and reference, respectively. Additional electrodes positioned on supra- and suborbital ridges and on external canthi were used to record electro-oculographic (EOG) activity generated by blinks/eye movements. Electrical impedances were maintained below 5 kΩ. Electrical signals were acquired with a Brain Vision V-Amp 16-channel amplifier and Brain Vision Recorder software (Brain Products, Munich, Germany), with amplifier bandpass filter settings at 0.1 to 100 Hz and a digital sampling rate at 500 Hz.

*Processing:* Off-line signal processing was performed using Brain Vision Analyzer® software (Brain Products, Munich, Germany). EEG recordings were inspected visually to eliminate segments that included head and eye movements as well as cardiac and muscle artifacts. EEG was segmented into 1100 ms epochs, beginning with 250 ms prestimulus (train) onset. Automatic artifact rejection followed, with epochs containing signals > 100 µV being eliminated. The remaining epochs were ocular corrected.^66^

Using a previously employed methodology, time–frequency analysis was performed using a Morlet wavelet transform with a constant (*c  *=*  σf/f*, where *σf*  =  the standard deviation of the center frequency [*f*] value of 15); this was computed, in 1-Hz frequency steps over the 20 to 50 Hz frequency range for estimation of gamma ERSP and over the 1 to 20 Hz range for estimation of theta ERSP. Specifically, evoked average power for the ASSR was defined within the 250 to 500 ms interval and was limited to the 37 to 43 Hz frequency range for gamma power and the 4 to 8 Hz frequency range for theta power. Power in both theta and gamma frequencies was quantified relative to average prestimulus activity, which was subtracted from each time point (in the 250-500 ms interval) for each frequency band.

PLF measures the phase variance of frequency-specific oscillations across single trials. Using a previous methodology,^15^ the PLF for theta and gamma frequencies was calculated as 1 – minus the circular variance of phases, and ranges from 0 (random distribution of phases) to 1 (perfect phase locking).^67^

Theta/gamma PAC was exported from preprocessed data and was imported into Brainstorm (v. 3.4 180725). For maximum coupling (maximum value within 4-8 Hz, 37-43 Hz), average coupling (average value between 4-8 Hz, 37-43 Hz), and each 1-Hz cross-frequency pairing (4/38; 5/38; 6/38; 7/38; 8/38; 4/40; 5/40; 6/40; 7/40; 8/40; 4/42; 5/42; 6/42; 7/42; 8/42 Hz) a modulation index value (ie, the strength of the PAC) was computed for the time period of 250-500 ms and exported for further statistical processing.

### Subjective Ratings

Participants completed an adverse events questionnaire requiring ratings on a scale of 1 to 5 of how they felt during the stimulation (1 being no symptoms at all and 5 being severe symptoms such as tingling, burning, itching, and headache). They were also asked whether they believed they had received an active or sham tACS treatment in order to confirm blindness to the treatments. This required that they select one of 3 options (I believe I had active tACS treatment; I believe I had the sham treatment; I am really not sure).

### Statistical Analysis

Data analysis was conducted using the Statistical Package for Social Sciences (SPSS; SPSS Inc., Chicago, IL). ASSR-dependent measures (ERSP, PLF, PAC) were analyzed with separate repeated-measures analysis of variance (ANOVA), with each ANOVA containing a 3-level within group treatment factor (sham tACS, theta tACS, and gamma tACS). For PAC, these ANOVAs were performed for each single (1 Hz) cross-frequency pairing in the theta–gamma bands. Similar ANOVAs were conducted for adverse events. Regardless of whether or not a significant main treatment effect was observed (*P* < .05), planned Bonferroni-adjusted comparisons were conducted between the 3 treatments.

## Results

Twenty-three participants (mean age  =  21.3  ±  1.9 SD) completed all 3 testing sessions and had usable EEG recording data.

### Auditory steady-state response and event-related spectral perturbation

**Figure 1.**
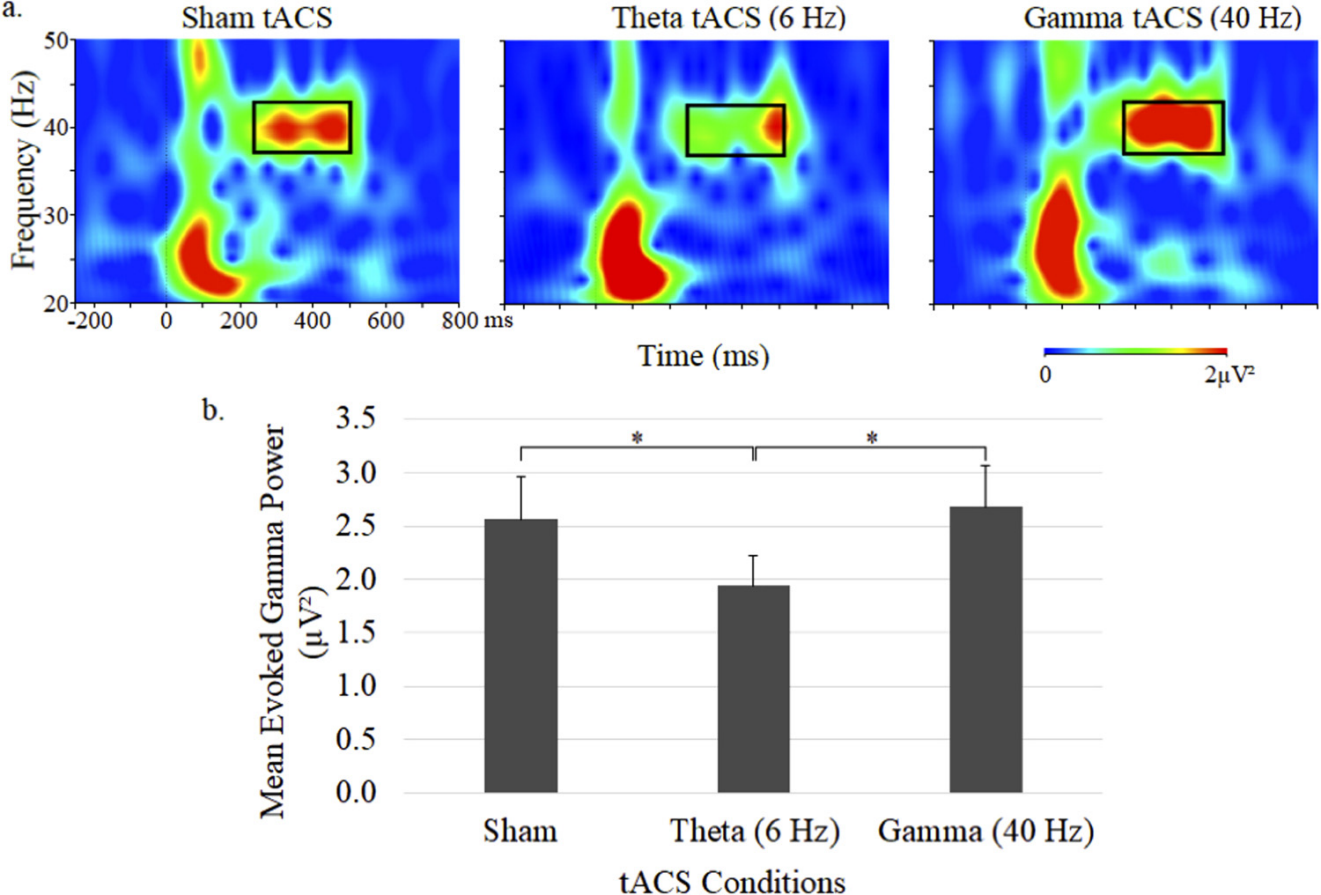
Grand average time–frequency plots (a) and mean (± SE) values (b) of the gamma ERSP at the central midline electrode site (Cz) under sham, 6-Hz and 40-Hz tACS conditions. In each map, the *x*-axis indicates the time (ms), beginning 250 ms preauditory stimulation, and the *y*-axis indicates the frequency, with the colour reflecting the ERSPs at each time–frequency point. Bar graphs display ERSP values during the 250 to 500 ms postauditory stimulation time interval.

Abbreviations: ERSP, event-related spectral perturbation; tACS, transcranial alternating current stimulation.

Time–frequency plots of gamma power and mean (± SE) ERSP for the 3 tACS sessions are shown in Figure 1. The 40-Hz auditory stimulus trains elicited gamma activity primarily within the 37 to 43 Hz range, which was found to be significantly influenced by tACS treatment (*F*  =  3.68, *df * =  2,44, *P*  =  .03). Follow-up comparisons between stimulation conditions showed a significantly reduced ERSP with theta tACS compared to both sham tACS (*P* < .005) and gamma tACS (*P* < .05), but no ERSP changes were found with gamma tACS relative to sham tACS.

**Figure 2.**
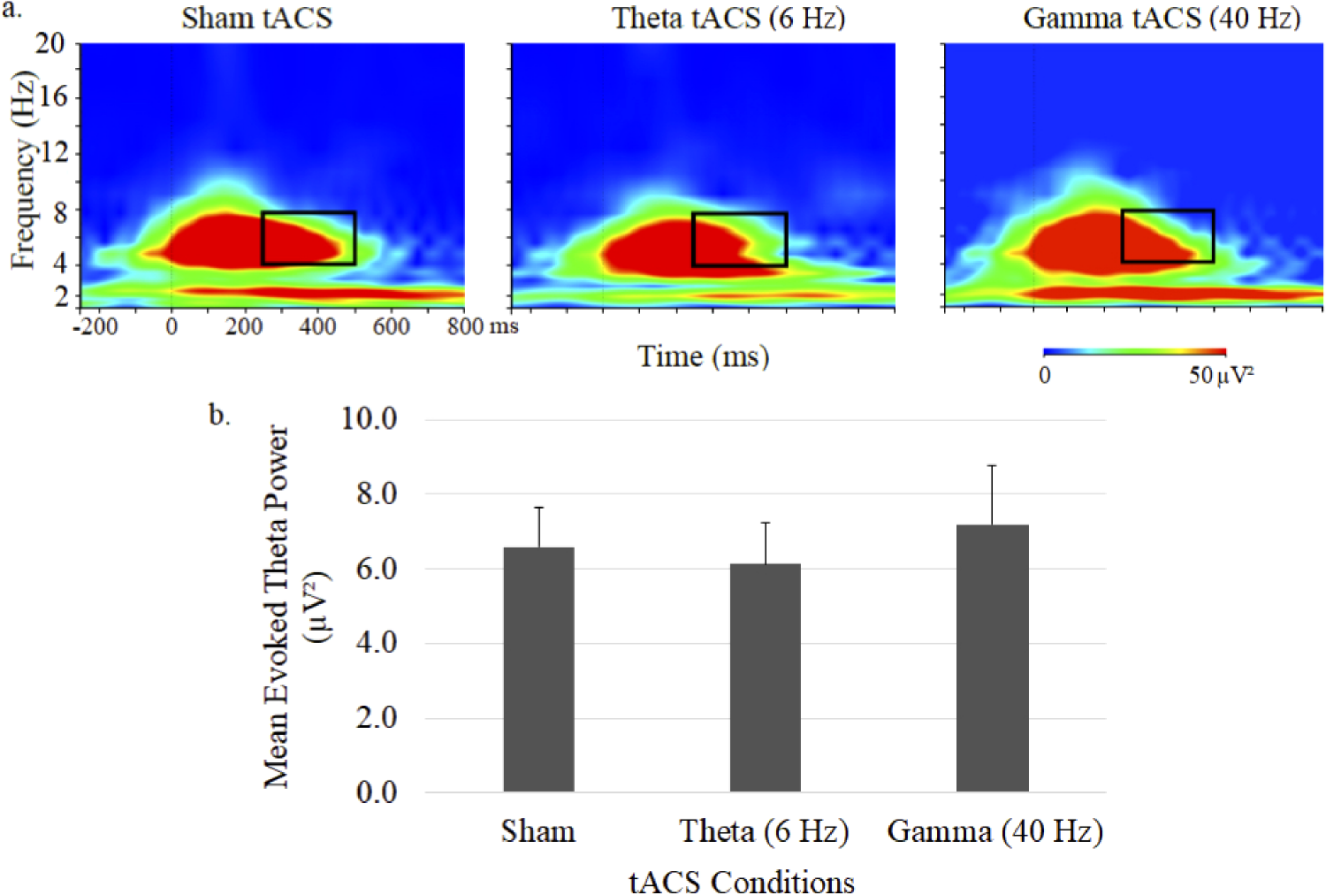
Grand average time–frequency plots (a) and mean (± SE) values (b) of the theta ERSP at the central midline electrode site (Cz) under sham, 6-Hz and 40-Hz tACS conditions. In each map, the *x*-axis indicates the time (ms), beginning 250 ms preauditory stimulation, and the *y*-axis indicates the frequency, with the colour reflecting the ERSPs at each time–frequency point. Bar graphs display ERSP values during the 250 to 500 ms postauditory stimulation time interval.

Abbreviations: ERSP, event-related spectral perturbation; tACS, transcranial alternating current stimulation.

Time–frequency plots of evoked theta power and mean (± SE) ERSP are shown in Figure 2. Theta ERSP during 40-Hz auditory stimulation was primarily evident in the 4 to 8 Hz range and showed no significant differences between the 3 tACS conditions.

### Auditory steady-state response and phase locking factor

**Figure 3.**
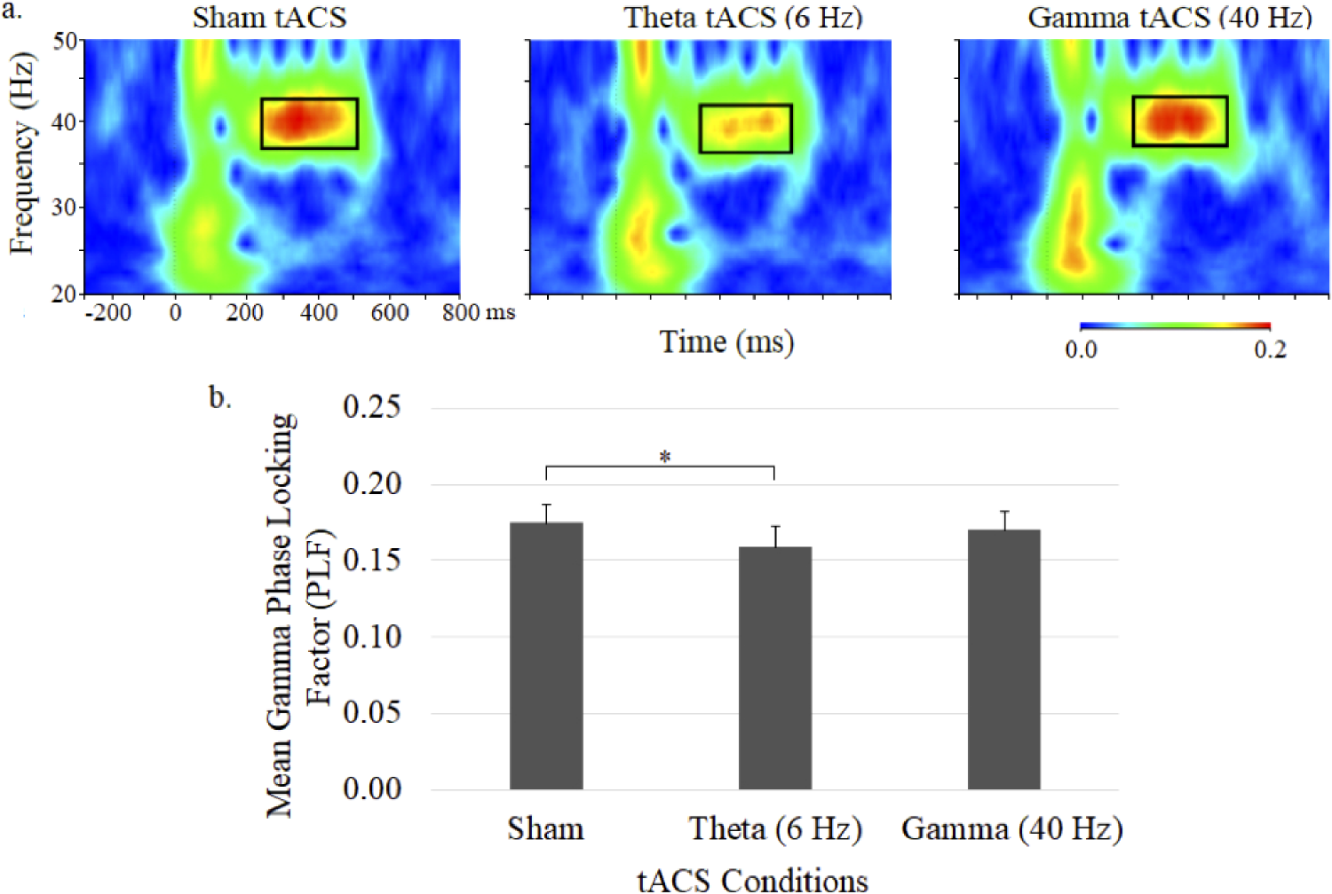
Grand average time–frequency plots (a) and mean (± SE) values (b) of the gamma PLF at the central midline electrode site (Cz) under sham, 6-Hz and 40-Hz tACS conditions. In each map, the *x*-axis indicates the time (ms), beginning 250 ms preauditory stimulation, and the *y*-axis indicates the frequency, with the colour reflecting the PLFs at each time–frequency point. Bar graphs display PLF values during the 250 to 500 ms postauditory stimulation time interval.

Abbreviations: PLF, phase locking factor; tACS, transcranial alternating current stimulation.

ASSR gamma phase as depicted in time-frequency plots for the 3 tACS conditions and mean gamma PLFs (± SE) are shown in Figure 3. Although there was no significant main effect for treatment, planned comparisons found a significantly diminished (*P* < .05) gamma PLF during theta tACS compared to sham tACS (Figure 3). Gamma tACS failed to alter gamma PLF.

**Figure 4.**
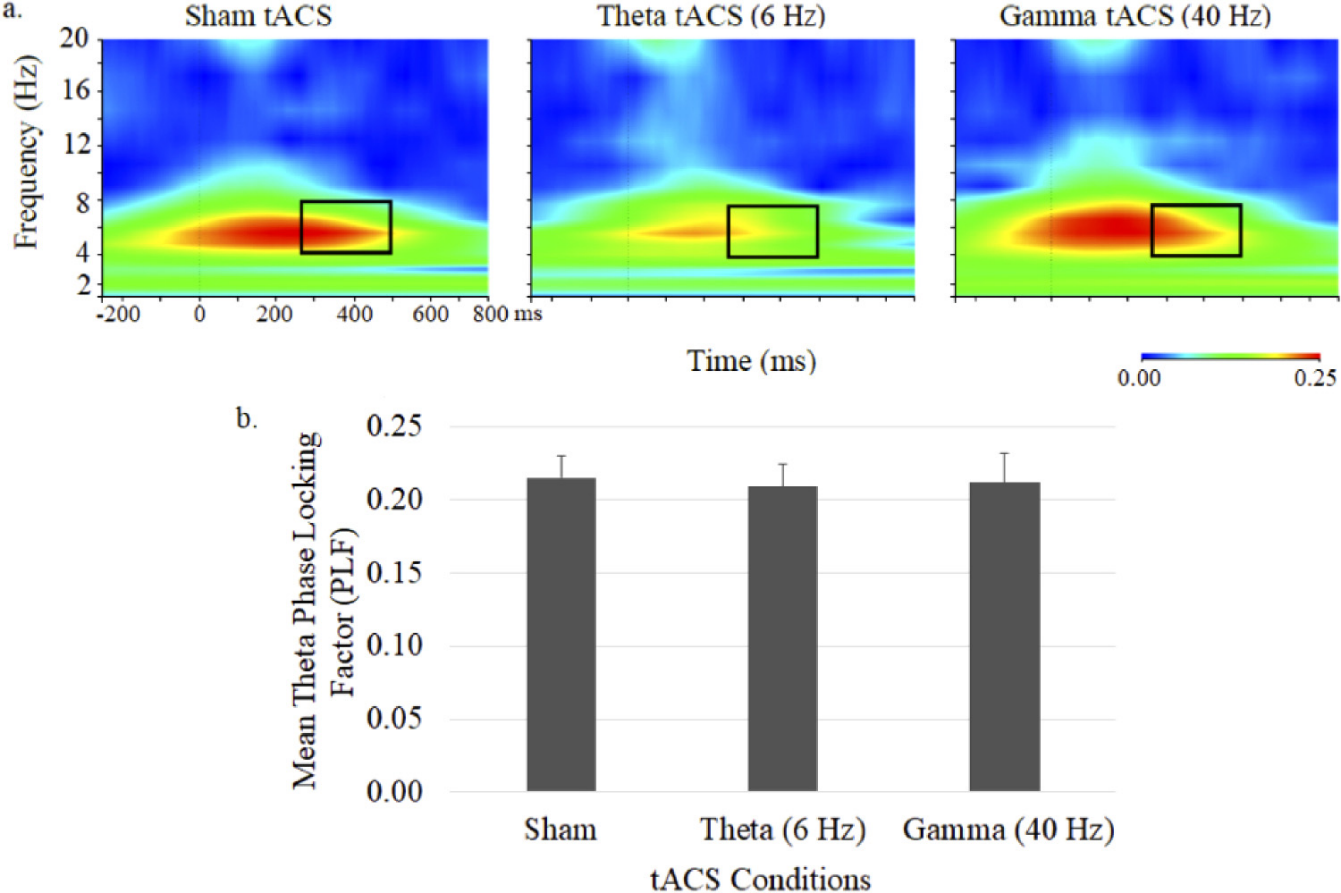
Grand average time–frequency plots (a) and mean (± SE) values (b) of the theta PLF at the central midline electrode site (Cz) under sham, 6-Hz and 40-Hz tACS conditions. In each map, the *x*-axis indicates the time (ms), beginning 250 ms preauditory stimulation, and the *y*-axis indicates the frequency, with the colour reflecting the PLFs at each time–frequency point. Bar graphs display PLF values during the 250 to 500 ms postauditory stimulation time interval.

Abbreviations: PLF, phase locking factor; tACS, transcranial alternating current stimulation.

Theta phase during 40-Hz auditory stimulation, shown in the time–frequency plots and mean (± SE) theta PLFs shown in Figure 4, appeared to be reduced during theta tACS but did not significantly differ across the 3 tACS conditions.

### Auditory steady-state response and phase–amplitude cross-frequency coupling

**Figure 5.**
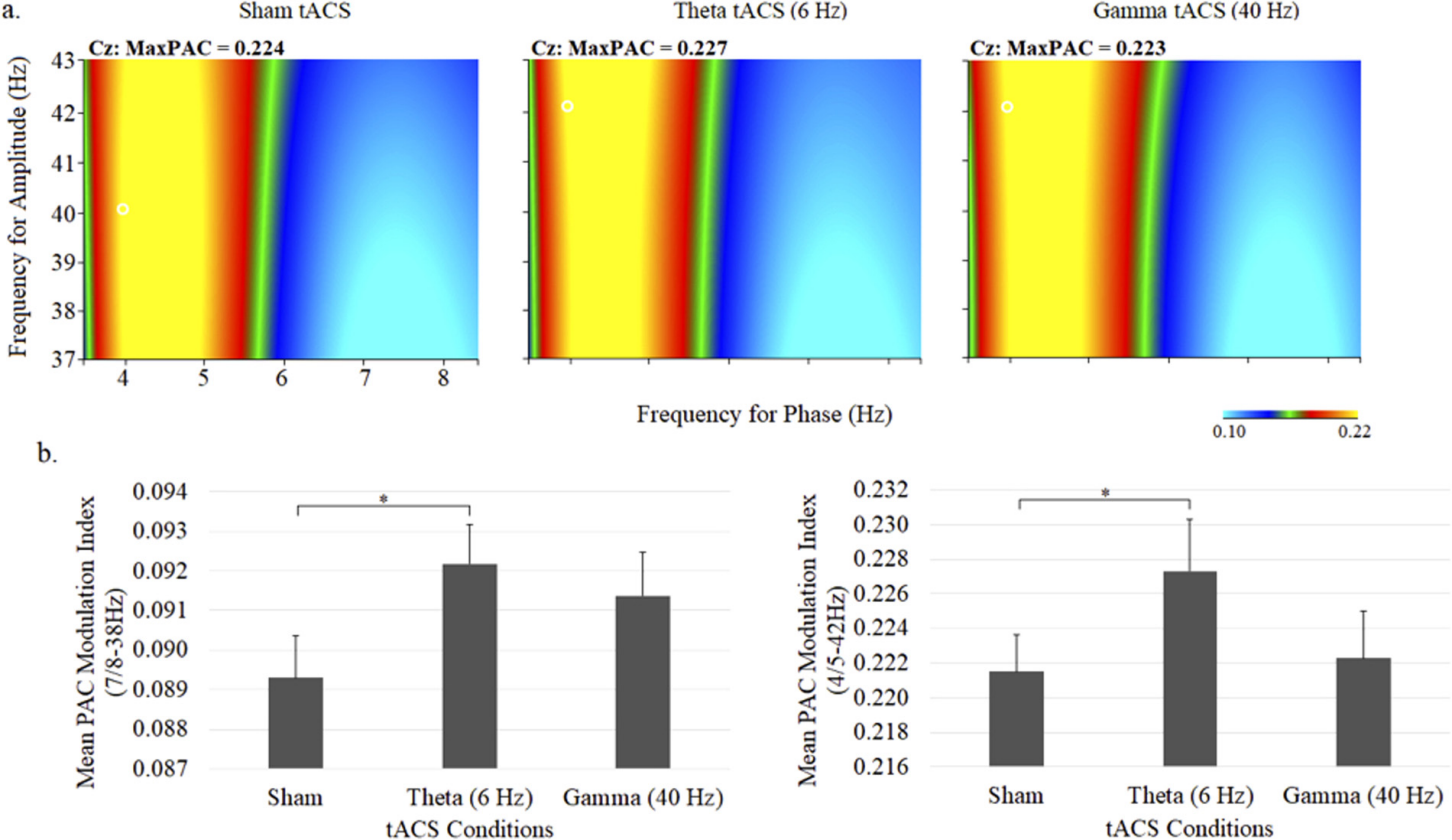
Grand average comodulograms portraying theta–gamma PAC (a) and mean (± SE) theta–gamma PAC values at the central midline electrode site (Cz) under sham, 6-Hz and 40-Hz tACS conditions. In each map, the *x*-axis indicates the frequency for phase (Hz) and the *y*-axis indicates the frequency for amplitude (Hz), with the colour reflecting the strength of the comodulation at each phase–amplitude point. Bar graphs display the PAC modulation indices for 7/8 to 38 Hz (left) and 4/5 to 42 Hz (right).

Abbreviations: PAC, phase-amplitude cross-frequency coupling; tACS, transcranial alternating current stimulation.

Comodulograms portraying theta phase – gamma amplitude coupling and mean (± SE) theta–gamma PAC values are shown in Figure 5. There were no main effects of stimulation or PAC pair, however, a main interaction effect was observed (stimulation × PAC pair: *F*  =  1.52, *df*  =  28,616, *P*  =  .04). Follow-up comparisons between stimulation conditions showed significantly increased PAC with theta tACS compared to sham tACS for the 7/38 Hz (*P* < .05), 8/38 Hz (*P* < .05), 4/42 Hz (*P* < .04), and 5/32 Hz (*P* < .04) PAC pairs. No differences were observed for gamma tACS.

#### Subjective Ratings

Across the 3 treatment conditions, participants were not able to accurately identify the stimulation they were exposed to. Although this suggested that the volunteers were blind to tACS treatment, mild adverse events (itching and tingling) were associated with gamma tACS compared to sham tACS (*P* < .001). These symptoms were not observed with theta tACS and none of the treatments were associated with moderately severe or severe adverse events.

## Discussion

In this study, we applied gamma and theta tACS over the bilateral temporal cortex and assessed their offline effects on auditory sensory processing with the oscillatory 40-Hz ASSR. Although not observed with high frequency (40-Hz) tACS, low frequency (6-Hz) tACS significantly diminished 40-Hz ASSR power and phase locking of gamma but not theta oscillations during 40-Hz auditory stimulation. Paralleling these ERSP and PLF changes with theta tACS were frequency-specific increases in theta-gamma PAC in response to 40-Hz auditory steady-state stimuli. These ERSP, PLF, and PAC observations suggest that neuromodulation with this brain electrical stimulation technique may be a promising approach for understanding neural oscillatory processing and for moderating neuronal reactivity deficits in the auditory system of schizophrenia patients.

Oscillatory activation patterns are fundamental in auditory sensation/perception,^58,68,69^ and accordingly, modulation of these intrinsic oscillation patterns by external stimulations should alter auditory processing. Transcranially applied alternating current in the alpha range has been shown in previous studies to reduce 40-Hz ASSR power in healthy volunteers.^49,50^ The putative mechanism for this observed tACS-induced effect is the entrainment of auditory alpha activity, which has been confirmed with findings of increased resting state power in the alpha band after tACS (an offline effect).^70^ Accounting for the increased task-induced suppression of ASSR by alpha tACS, alpha oscillations have been interpreted as markers for increased inhibition in multiple sensory domains including the auditory modality.^71,72^ This view is reinforced by findings of nonlinear interactions,^73^ and inverse relationships between the ASSR and intrinsic alpha oscillations.^74^

In the only 2 studies to examine the modulatory effects of nonalpha tACS on 40-Hz ASSR, gamma (40-Hz) tACS increased ERSP and intertrial phase coherence (ITPC),^45,48–50^ while theta (6.5 Hz) tACS produced a reduction in ASSR gamma power, which was similar to that seen with alpha tACS, but in one study was not significantly different from no tACS.^49^ Failure to observe theta tACS effects in this latter study may be related to differences in tACS methodology as theta stimulation was only applied for 6 min (vs 20 min in our procedure) and unlike our study which applied electrical stimulation at individual thresholds, each participant was administered the same dose (1.5 mA). In this present study, gamma tACS failed to change ASSR ERSP or PLF in either theta or gamma frequencies, but tACS in our study was administered at a higher intensity (2 mA vs 1.5 mA) and for a longer duration (20 min vs 10 min) than in the previous work.^48–50^ Although not affecting theta ERSP or PLF, we observed that tACS did diminish ASSR and resulted in a schizophrenia-like ASSR, diminishing both ERSP and PLF. NMDAR hypofunctioning is a core mechanism of schizophrenia. Given that the 40-Hz ASSR is a pharmacodynamic biomarker of cortical NMDARs,^14,21–24^ with NMDAR antagonists disrupting theta modulation of gamma,^75,76^ these present findings indirectly suggest that theta tACS effects on gamma ASSR are mediated in part by alterations in glutamatergic neurotransmission.

Direct evidence that theta tACS may have acted to modulate the coupling of auditory-evoked gamma power to the theta rhythm is shown by frequency-specific alterations in theta–gamma PAC, which were found not to be induced with gamma tACS. In the only other tACS study to examine cross-frequency coupling during ASSR, alpha tACS failed to alter the coupling of ASSR gamma power to the dominant alpha frequency.^44,45,48–50^ Although not observed in earlier ASSR studies,^18,77^ one recent investigation reported higher theta-gamma PAC during 40-Hz ASSR in patients with schizophrenia^78^ while another study showed intact ASSR-related PAC across phases of the illness.^79^ Gamma amplitude (power) has been shown to be modulated by theta phase in scalp EEG,^57,80^ intracranial EEG,^81,82^ and local field potential studies,^58,83^ as well as in several brain regions including the cerebral cortex,^57^ hippocampus,^80^ and auditory cortex^58^ which are areas also involved in ASSR generation.^18,81,82^ The hierarchical coordination of low- and high-frequency neural oscillations serve an important role in integrating sensory and cognitive information^83^ and their offline entrainment by tACS, lasting for up to 1 h poststimulation,^84^ is believed to result in lasting functional changes mediated by alterations in spike-timing-dependent plasticity involving NMDAR activity.^85^ Given that disrupted synchronous gamma oscillations have been identified as a potentially important biomarker for sensory and cognitive symptoms in schizophrenia that are linked to NMDAR-moderated cortical plasticity deficits,^86^ theta tACS neuromodulation of 40-Hz ASSR-related theta–gamma PAC may be a useful approach for furthering our understanding of gamma disruptions and for initiating and sustaining lasting changes in auditory sensory processing.^87^

### Limitations

Although these preliminary results suggest that theta tACS may be a potentially useful noninvasive brain stimulation tool for investigating neuromodulatory mechanisms regulating 40-Hz ASSR and for targeting 40-Hz ASSR deficits in schizophrenia, a number of limitations require addressing. First, the sample size was relatively small and may have lacked statistical power to detect the full complement of tACS effects. Related to this issue is the problem that the sample only consisted of males and as such the results are not generalizable to the general population. Second, stimulation parameters for both theta and gamma tACS were fixed with respect to frequency, intensity, duration and site of stimulation, and as previous tACS studies have shown that stimulation effects depend on such parameters, they require investigating in future theta tACS work. These investigations should also compare different tACS frequencies to determine the specificity of theta tACS. Additionally, only acute single-session treatment effects were examined and, as in tACS studies in patients with schizophrenia^38,43–45,48–50^ multisession effects of tACS require attention. Complimenting EEG measures in these studies with tests of sensory of cognitive tasks would provide insight into the functional significance of 40-Hz ASSR alterations under tACS. Finally, although assessment of the offline effects of tACS has potential importance for the clinical application of theta tACS, studying the online effects of theta tACS with concurrent high-density EEG monitoring and source reconstruction techniques would be helpful in furthering our mechanistic understanding of the network dynamics of this brain stimulation technique.^55^

## Conclusion

These study findings tentatively support the use of tACS as a noninvasive technique for modulating sensory cortical gamma synchronization by low-frequency sinusoidal waveforms. Via its influence over power, phase locking and cross-frequency coupling, tACS may be useful for understanding mechanisms of action of cortical oscillatory dynamics in normal and disturbed auditory sensory processing.
